# Supplementary material for: Ecological momentary assessment of physical and eating behaviours: The WEALTH feasibility and optimisation study with recommendations for large-scale data collection
Source: PLoS One. 2025 Feb 11;20(2):e0318772. doi: 10.1371/journal.pone.0318772 (PMC11813119; doi:10.1371/journal.pone.0318772)
Supplement: S1 File — (PDF) [file pone.0318772.s001.pdf]

Topic guide for feedback interviews (WEALTH)

1. Smartphone

⇒ Did you use your own smartphone or a loaned study smartphone?

⇒ If you used your own smartphone, what was its brand and model, and what was the version of the operating system (i.e., iOS 16.0.2 or Android 11)?

- **Notifications.** Did you have any issues with the notifications?
- **Missed questionnaires.** Have there been situations where you didn't find out about the survey even though you were within range of the phone and didn't have it completely silenced? How do you explain this?
- **Unanswered questionnaires.** On what occasions have you been unable to respond to a request to complete a questionnaire even though you were aware of it? On average, how many times a day did this happen?
- **Inconvenience**. On what occasions did you find completing the questionnaire inconvenient (bothersome/uncomfortable/inappropriate)? How did you deal with it?
- ***Study phone.*** *Only for participants with a study phone:* If you were to participate in a similar study again, would you prefer to use your personal phone for the study? If yes, why? Did carrying a second phone cause you any difficulties or complications?
- **HealthReact app.** Do you have any insights or recommendations about the HealthReact app?

2. Questionnaires

- **Number of questionnaires.** In retrospect, how do you perceive the number of questionnaires you completed daily? *If they are unsure how to answer, offer them possible answers: I could do more, it was just enough, or too much.*
- **Length of the questionnaires.** In retrospect, how do you perceive the length of the questionnaires you filled out? *If they are unsure how to answer, offer them possible answers: it could be longer, just right, or too long.*

⇒ Would you complete more questionnaires if the number of questions per questionnaire was reduced?

⇒ Would you be willing to complete the same number of questionnaires if the number of questions per questionnaire increased?

- **Morning/evening questionnaires.** Did the first morning or last evening questionnaire conflict with your sleep pattern? What adjustments would you suggest?
- **Self-initiated meal/snack/drink.** How did you manage to report every meal/snack/drink you consumed? What percentage of drinks/meals/snacks did you fail to report?

⇒ What was the usual time delay from meal/snack/drink when you reported it?

3. Questions

- **Troubling questions.** Did any questions cause you difficulties, e.g., due to ambiguity, lack of clarity or missing answer options? Which ones? How would you suggest modifying them?
    - *Have a printed questionnaire ready and, if necessary, allow the participant to refer to it to recall the question. However, there is no need to go through all the questions systematically.*

4. SACANA dietary recall

- Were there any technical issues when you completed SACANA? Did you face any difficulties in entering your food and drink items?

5. General

- **Training.** How would you rate the initial training for the study? How would you rate the materials you received for the study? Was there any information you missed?
- **Technical issues and support.** Did you encounter any technical difficulties during the study? What were they? Did you receive sufficient support?

⇒ How many times would you have contacted technical support during the study if it had been available?

⇒ If you were to participate in similar research in the future, what would you consider adequate compensation (in EUR) for your time spent in this study?

⇒ Overall, how would you rate your experience in this study on a scale of 1 - 10, where (1) very negative (I would not participate in something like this again) and (10) very positive (I would definitely do it again)?
