# Supplementary material for: Ecological momentary assessment of physical and eating behaviours: The WEALTH feasibility and optimisation study with recommendations for large-scale data collection
Source: PLoS One. 2025 Feb 11;20(2):e0318772. doi: 10.1371/journal.pone.0318772 (PMC11813119; doi:10.1371/journal.pone.0318772)
Supplement: S2 File — (PDF) [file pone.0318772.s002.pdf]

EMA SURVEYS

1. Time-based surveys

a) Daily surveys (9 items)

	Item	Response
**1**	What were you doing right before the prompt?	(1) Consumed drink, snack or meal, (2) Watched TV, (3) Used a computer/smartphone, (4) Read (5) Played a game, (6) Was in transport (car, bus, train), (7) Was on the move (walking, shopping, etc.), (8) Exercised, (9) Socializing (being with friends/family) (10) Other
**2**	How do you feel right NOW?	"Very tired" – "Very awake"
**3**	How do you feel right NOW?	"Very content" – "Very discontent"
**4**	How do you feel right NOW?	"Very agitated" – "Very calm"
**5**	How do you feel right NOW?	"Very full of energy" – "Very without energy"
**6**	How do you feel right NOW?	"Very unwell" – "Very well"
**7**	How do you feel right NOW?	"Very relaxed" – "Very tense"
**8**	How would you rate your fatigue level right NOW?	"No fatigue" – "Extreme fatigue"
**9**	I have an intense desire to eat one or more specific foods.	(1) strongly disagree, (2) disagree, (3) neutral, (4) agree, (5) strongly agree

b) Morning surveys (same 9 items as the daily surveys plus 5 additional items to assess sleep duration and quality)

	Item	Response
**1**	At what time did you go to bed yesterday? Indicate in hours and minutes format, e.g.,22:15.	HH:MM (00:00)
**2**	How long did it take for you to fall asleep last night? Indicate amount of time in hours and minutes format, e.g., 00h 20min	HH:MM (00:00)
**3**	What time did you wake up this morning? Indicate in hours and minutes format, e.g., 06:15.	HH:MM (00:00)
**4**	How many hours and minutes did you actually sleep last night? (This may differ from the number of hours spent in bed) Indicate amount of time in hours and minutes format, e.g., 07h 45min	HH:MM (00:00)
**5**	How would you rate your sleep quality last night?	"Very bad" – "Very good"

c) Evening surveys (same 9 items as the daily surveys plus 5 additional items to assess general well-being)

	Item	Response
**1**	I have felt cheerful and in good spirits.	(0) at no time, (1) some of the time, (2) less than half of the time, (3) more than half of the time, (4) most of the time, (5) all the time
**2**	I have felt calm and relaxed.	(0) at no time, (1) some of the time, (2) less than half of the time, (3) more than half of the time, (4) most of the time, (5) all the time
**3**	I have felt active and vigorous.	(0) at no time, (1) some of the time, (2) less than half of the time, (3) more than half of the time, (4) most of the time, (5) all the time
**4**	I woke up feeling fresh and rested.	(0) at no time, (1) some of the time, (2) less than half of the time, (3) more than half of the time, (4) most of the time, (5) all the time
**5**	My day has been filled with things that interest me.	(0) at no time, (1) some of the time, (2) less than half of the time, (3) more than half of the time, (4) most of the time, (5) all the time

2. Event-based surveys

a) Sedentary surveys (same 9 items as the daily surveys plus 5 additional items covering body posture, physical behaviour, location, and company)

	Item	Response
**1**	What was your body position right before the prompt?	(1) Lying, (2) Reclining, (3) Sitting, (4) Standing, (5) Kneeling
**2**	What physical action were you performing right before the prompt?	(1) Walking, (2) Running, (3) Cycling, (4) Exercising, (5) Other
**3**	Where are you right NOW?	(1) Indoor<br>(2) Outdoor
**4a**	Where indoor are you right now?	(1) At home, (2) At someone else´s home, (3) At a restaurant, café, pub; (4) At (snack-) bar, (5) At school or work, (6) On the move (car, bus, etc.), (7) Shopping centre, mall, market, shop, (8) At a sport or entertainment venue, (9) Somewhere else
**4b**	Where outdoor are you right now?	(1) In a domestic garden, (2) On the street, (3) In a park /urban green space, (4) On the move (car, bus, etc.), (5) Outdoor sports fields, courts, (6) Country road (with traffic), (7) Countryside (incl. forest, beach, etc.), (8) Somewhere else
**5**	Who are you with right NOW?	(1) *Alone, (2) With friends/classmates/ colleagues, (3) with family, (4) with children, (5) with strangers, (6) with a crowd, (7) with an organized group, (8) other

*no other item can be selected when this is selected

b) Walking surveys (same 9 items as the daily surveys plus 8 additional items covering body posture, type and intensity of physical activity, location, and company)

	Item	Response
**1**	What was your body position right before the prompt?	(1) Lying, (2) Reclining, (3) Sitting, (4) Standing, (5) Kneeling
**2**	What physical action were you performing right before the prompt?	(1) Walking, (2) Running, (3) Cycling, (4) Exercising, (5) Other
**3**	What was the nature of the activity you were doing?	(1) Moving on flat surface, (2) Ascending a slope/stairs, (3) Descending a slope/stairs, (4) Moving without free arm swing (e.g., holding a child), (5) Moving while carrying a load (e.g., shopping bag)
**4**	What was the pace of the walk/run you were doing?	(1) Slow, (2) Steady/average, (3) Brisk pace
**5**	What was the intensity of the activity you were doing?	(1) Very light activity, (2) Light activity, (3) Moderate activity, (4) Vigorous activity, (5) Very hard activity, (6) Maximal effort activity
**6**	Where are you right NOW?	(1) Indoor<br>(2) Outdoor
**7a**	Where indoor are you right now?	(1) At home, (2) At someone else´s home, (3) At a restaurant, café, pub; (4) At (snack-) bar, (5) At school or work, (6) On the move (car, bus, etc.), (7) Shopping centre, mall, market, shop, (8) At a sport or entertainment venue, (9) Somewhere else
**7b**	Where outdoor are you right now?	(1) In a domestic garden, (2) On the street, (3) In a park /urban green space, (4) On the move (car, bus, etc.), (5) Outdoor sports fields, courts, (6) Country road (with traffic), (7) Countryside (incl. forest, beach, etc.), (8) Somewhere else
**8**	Who are you with right NOW?	(1) *Alone, (2) With friends/classmates/ colleagues, (3) with family, (4) with children, (5) with strangers, (6) with a crowd, (7) with an organized group, (8) other

3. Meals, snacks, and drinks surveys

Self-initiated surveys (8 items covering the drink or snack category, location, company, and concurrent and preceding behaviour)

	Item	Response
**1**	What do you want to report?	(1) Breakfast, (2) Lunch, (3) Dinner, (4) Snack, (5) Drink
**2**	Please enter the time when you consumed this drink/meal/snack.	HH:MM (00:00)
**3a**	Please select what you consumed as your snack.	(1)Nuts and seeds (e.g. <local examples> etc.), (2)Dried or fresh fruits (e.g. <local examples> etc.), (3)Snacks like crisps, corn crisps, popcorn etc. (e.g. <local examples> etc.), (4)Snacks like savoury pastries and fritters (e.g. crackers cheese pie, sausage pie, pancakes,<local examples> etc.), (5)Snacks like chocolate, candy bars (mars, lions, kit kat,<local examples> etc.), (6)Snacks like candies, loose candies, marshmallow (e.g. <local examples> etc.), (7)Snacks like biscuits, packaged cakes, or pastries and puddings (e.g. like sweet bakery products<local examples> etc.), (8)Ice cream, milk or fruit-based bars (<local examples> etc.), (9) Hamburger, hot dog, kebab, wrap, falafel, sandwiches (<local examples > etc.), (10) Other snack
**3b**	Please select what you consumed as your drink.	(1) Water (tap water, carbonated water, etc.), (2) Fruit juices (100% fruit,etc.), (3) Carbonated sugar sweetened drinks (cola, fanta, etc.), (4) Artificially sweetened drinks (diet cola, etc.), (5) Sugar sweetened drinks (iced teas, fruit juices with less than 100% juice, energy drinks, teas/coffee with milk or sugar), (6) Coffee/tea without milk or sugar, (7) Alcoholic drinks, (8) Else
**4**	Where were you when you consumed this drink/meal/snack?	(1) Indoor, (2) Outdoor
**5a**	Where indoor were you when you consumed this drink/meal/snack?	(1) At home, (2) At someone else´s home, (3) At a restaurant, café, pub, (4) At (snack-) bar, (5) At school or work, (6) On the move (car, bus, etc.), (7) Shopping centre, mall, market, shop, (8) At a sport or entertainment venue, (9) Somewhere else
**5b**	Where outdoor were you when you consumed this drink/meal/snack?	(1) In a domestic garden, (2) On the street, (3) In a park /urban green space, (4) On the move (car, bus, etc.), (5) Outdoor sports fields, courts, (6) Country road (with traffic), (7) Countryside (incl. forest, beach, etc.), (8) Somewhere else
**6**	Who were you with when you consumed this drink/meal/snack?	(1) *Alone, (2) With friends / classmates / colleagues, (3) With family , (4) With children, (5) With strangers, (6) With a crowd, (7) With an organized group, (8) Other
**7**	What were you doing while consuming this drink/meal/snack?	(1) Consumed drink, snack or meal , (2) Watched TV, (3) Used a computer/smartphone, (4) Read, (5) Played a game, (6) Was in transport (car, bus, train), (7) Was on the move (walking, shopping,etc.), (7) Exercised, (8) Socializing (being with friends/family), (8) Other
**8**	What were you doing in the hour prior to consuming this drink/meal/snack?	(1) Consumed drink, snack or meal, (2) Watched TV, (3) Used a computer/smartphone, (4) Read, (5) Played a game, (6) Was in transport (car, bus, train), (7) Was on the move (walking, shopping,etc.), (8) Exercised, (9) Socializing (being with friends/family), (10) Other
