# Supplementary material for: Ecological momentary assessment of physical and eating behaviours: The WEALTH feasibility and optimisation study with recommendations for large-scale data collection
Source: PLoS One. 2025 Feb 11;20(2):e0318772. doi: 10.1371/journal.pone.0318772 (PMC11813119; doi:10.1371/journal.pone.0318772)
Supplement: Attachment — Submitted filename: Response letter.docx [file pone.0318772.s003.docx]

PONE-D-24-30072

Ecological momentary assessment of physical and eating behaviours: the WEALTH feasibility and optimisation study with recommendations for large-scale data collection

PLOS ONE

I have reviewed your manuscript "Ecological momentary assessment of physical and eating behaviours: the WEALTH feasibility and optimisation study with recommendations for large-scale data collection" and the comments from three reviewers. The reviewers found your manuscript to be thorough, well-written, and valuable for the field. They particularly appreciated your novel approach using Fitbit for event-triggered EMA and your clear recommendations for future research.
The reviewers have suggested some minor revisions that would strengthen the manuscript.
Given that these revisions are relatively straightforward and do not require major restructuring, I recommend a minor revision.

Dear Dr. Shevchenko,

Thank you for the opportunity to revise our manuscript and for facilitating the thoughtful feedback from the reviewers. We have carefully addressed all comments and made the necessary changes to enhance the clarity and comprehensiveness of the paper.

**Reviewer #1:**

The submitted manuscript is a thorough investigation of the feasibility of an intensive EMA protocol measuring eating, drinking, walking, and sedentary bouts via both self-report surveys and wearable device. The authors use a combination of Fitbit and self-report surveys to identify physical activity objectively and prompt users to report on their physical activity in event-based surveys. The manuscript is refreshingly neutral and straightforward about the authors’ findings on feasibility, with helpful details on methodology and constructive suggestions for replicating these methods and applying these findings to future investigations. The methods employed are varied, sometimes without clear rationale, and more clarity is needed on what exactly was measured within the EMA surveys.
>>> Thank you for your thoughtful and positive feedback. We appreciate your recognition of our efforts to present a thorough investigation with transparent findings and constructive suggestions. Your comments are greatly encouraging.

Abstract:
It would aid reader understanding to specify what was being measured in the EMA protocol (that is, what physical and eating behaviors?)

>>> Thank you for this suggestion. To enhance clarity, we have extended the relevant sentence in the abstract to specify what was being measured in the EMA protocol. The revised text now reads:

*"The EMA protocol included time-based (7/day), event-based (up to 10/day), and self-initiated surveys, each containing 8 to 17 items assessing physical and eating behaviours and related contextual factors such as affective states, location, and company."* *[lines 42 - 44]*

Introduction:
It is novel that the authors use Fitbit to identify events when an event-contingent survey is indicated. In many past studies, event-contingent EMAs are essentially self-initiated; a participant is told to initiate a survey when they do a particular activity, but there is usually no means to verify that they actually did. The authors’ use of Fitbit to address this weakness in EMA methodology is novel and worth additional emphasis.

>>> Thank you for appreciating the novelty of our approach. We have emphasised this aspect in the manuscript. The revised text reads:

*"Recent advances in sensor technologies have introduced the novel concept of event-based EMA, where surveys are dynamically triggered by specific events detected by sensors, offering a more context-aware alternative to traditional time-based approaches."* *[lines 97 - 100]*

When the authors review compliance rates found in prior literature, it should be specified if these are time-based or event-based assessment compliance rates. They were likely time-based, since the authors’ use of Fitbit to identify events and associated surveys is novel.

>>> Thank you for the comment. The majority of compliance rates cited were indeed from time-based assessments, except for the study summarising person-level data from four independent EMA studies [Compernolle et al., 2024], which used both time-based and sensor-triggered event-based prompting schedules. This information has been added to the manuscript as follows:

*"Furthermore, a recent study summarising person-level data from four independent EMA studies, which used both time-based and sensor-triggered event-based prompting schedules to explore physical and eating behaviours among 278 older adults, found a compliance rate of 75%, varying from 65% to 83% across four studies, with variations among participant subgroups and at different times of the day." [lines 125 - 129]*

Method:
The days are sometimes called Day 1, Day 2, etc. and other times Day -1, Day 0, etc.

>>> We have ensured that the days are referred to consistently throughout the manuscript and added the day numbers where they were missing. The updated text now reads:

*"The protocol consisted of 9 days of free-living EMA data collection (Figure 1). The first day (day -1) served as a training day in the lab, the second day was a lead-in day (day 0) where participants familiarized themselves with the study protocol and devices in their natural environment, and the data from the subsequent 7 days were included in the analyses (days 1 to 7)."* *[lines 292 - 296]*

We need more information about the content assessed within the surveys. What were the questions asked? Some more information is needed for readers to understand how similar their own protocols are to the one tested in this study.

>>> In the Methods section, we provided an overview of the surveys' content: "*The daily surveys included 9 items covering behaviour, affective states, fatigue and food cravings. ... The morning survey included the same items as the daily surveys plus 5 additional items to assess sleep duration and quality. ... The evening survey included the same items as the daily surveys plus 5 additional items to assess general well-being for the respective day.* *[lines 309 - 314] ... The sedentary surveys included the same items as the time-based daily surveys plus 4 additional items covering body posture, physical activity, location, and company.* *[lines 323 - 325] ... The walking surveys included the same items as the daily surveys plus 8 additional items covering body posture, type and intensity of physical activity, location, and company.* *[lines 335 - 337] ... The meals, snacks, and drinks surveys included 8 items covering the drink or snack category, location, company, and concurrent and preceding behaviour; however, participants were not asked to specify the components or foods of the meals, snacks, and drinks.*" *[lines 350 - 352]*

For readers who are interested in more details, we have now included a new supplementary file S2 containing the full list of survey questions. *[lines 313 - 314]*

Why did the activity (sedentary, walking, and running) surveys need to be done so closely to the actual event (1 or 2 minutes after, with only 8 minutes allowed before the survey expired)? Could the items not be assessed retrospectively (e.g., “when you were inactive recently…”)? Perhaps I would understand better if I knew exactly what was being measured in the surveys.

>>> Thank you for this comment. We designed the surveys to capture momentary states as close as possible to the event of interest to minimise recall bias and ensure the data reflect participants' experiences in real time. We hope that the inclusion of the full list of survey questions in the supplementary file provides greater clarity on what was being measured and the rationale for the timing of the surveys.

Results:
Am I understanding correctly that the authors aren’t reporting completion times for France and Ireland because the times would be variable depending on how many questions the participant answered affirmatively about meals/drinks? Given that many other researchers may include assessment of meals/drinks in their surveys in a similar way, I think it could still be valuable to report completion times if you have the data.

>>> Yes, that is correct. We did not report completion times for France and Ireland because these surveys might include reports on meals, snacks, and drinks. Furthermore, the reports on meals, snacks, and drinks had variable lengths due to branching based on participants' responses, making the completion times non-comparable. While we acknowledge that such data might be interesting for researchers with similar survey designs, this was not the primary objective of our study, and we therefore prefer not to include them.

Why was the optimal number of surveys 4/day for sedentary events and 3/day for walking events? With the 90 minute interval between them, it seems it would be difficult for participants to have that many in a day. To assess sedentary behavior 4x/day, the authors define a sedentary episode as no movement for only 20 minutes, which for many people with desk jobs is not very long. I can imagine participants being irritated by the implication that they are not moving enough after just 20 minutes of inactivity.

>>> The optimal number of surveys was determined by expert consensus within the WEALTH consortium. This decision was made to enable achieving the main objectives of the WEALTH project, which aimed to collect EMA-labeled accelerometry data from a large sample of healthy adults to apply machine learning methods and develop an integrated data collection system that simultaneously captures physical and eating behaviours and their interrelation. We have updated the manuscript for clarity:

*"During the development of the EMA protocol, the WEALTH team also determined the optimal number of event-based surveys through expert consensus to align with the project objectives of collecting a large dataset of EMA-labeled accelerometry data for the application of machine learning methods. Specifically, the optimal number of surveys was set at four per day for sedentary surveys and three per day for walking and running surveys, balancing the feasibility of collecting sufficient data with minimizing participant burden."* *[lines 285 - 281]*

We understand the concerns regarding the survey frequency and the 20-minute threshold for sedentary behaviour. That is precisely why we conducted this feasibility and optimisation study, which confirmed that the survey design and thresholds were acceptable for participants and feasible for data collection. Thank you for highlighting these points, and we are happy to provide further clarification if needed.

Discussion
In the section “improving participants’ compliance”, it is not mentioned that lengthening the time limit allowed for survey completion would also improve compliance.

>>> Thank you for your comment. We have clarified this point by explicitly stating that extending the expiration time could improve compliance by providing participants with greater flexibility to respond. The updated text now reads:

*"Furthermore, respecting participants’ feedback that the time window when the surveys were available for completion before they expired was too short, it was recommended that the expiration be extended from 8 to 15 minutes. Extending the expiration time could improve compliance by providing participants with greater flexibility to respond. However, this recommendation must balance providing sufficient time for participants to respond with the need to answer the survey close to the time of the trigger, which is especially important when the survey relates to specific events of interest."* *[lines 622 - 629]*

There is a missing reference at line 605.

>>> Thank you for pointing this out. We have completed the missing reference [Stamatakis et al., 2019].

Line 637 Another way of addressing the participants’ reasonable concern that reporting sips of water was overly burdensome is to ask participants to report when they start and finish a

drink, and assume that they were sipping it in between.

>>> Thank you for this suggestion. While asking participants to report when they start and finish a drink could reduce the reporting burden, this approach does not align with our aim to capture momentary states as close to the event as possible.

Limitations
I do think a noteworthy limitation of the study is the recruitment of a convenience sample of people personally familiar to the authors. The sample’s motivation to complete the surveys and the study in general could be different than a general research participant sample. For example, all participants completed the study, which is unusual and may be due to an interpersonal sense of obligation.

>>> Thank you for pointing this out. We have acknowledged this limitation in the manuscript and noted that the convenience sampling approach aligns with the recruitment strategy for the WEALTH project. The updated text now reads:

*"Finally, the recruitment of a convenience sample, including individuals personally familiar to the authors, may have influenced participants' motivation to complete the study and contributed to the unusually high retention rate due to a sense of interpersonal obligation.“* *[lines 714 - 718]*

I would also add that not fully measuring what was consumed in meals/drinks (if I understood the method correctly) makes this study a bit less helpful in understanding how burdensome and feasible it would be to actually measure food/drink consumed with an EMA protocol.

>>> Thank you for raising this valid point. However, measuring dietary intake and assessing the specific foods and drinks consumed is highly complex and is typically achieved through different methods, such as 24-hour dietary recalls or food frequency questionnaires, which were outside the scope of this study. Our focus was on evaluating the feasibility of the EMA protocol for capturing physical and eating behaviours and the context in which they take place in real time. For this reason, we prefer not to include an analysis of the burden and feasibility of detailed dietary tracking in this study. We appreciate your understanding.

**Reviewer #2:**

This study evaluates a pilot or feasibility study to optimize the study design for a large-scale EMA-study conducted in different European countries (WEALTH Project). It focused on the compliance rate and on the design of event- and time-based EMA assessments (sampling scheme). The paper deepens our understanding of some challenging issues (e.g. compliance with EMA protocol, managing the burden of frequent prompts) that are highly relevant to obtaining a reliable and credible dataset when conducting an EMA-study.
The paper is clear and provides useful information for the readers. It makes important points that are widely applicable. I just have a few issues – mainly for the discussion section – that need to be addressed before it’s ready for publication.

>>> Thank you for your positive and encouraging feedback. We appreciate your recognition of the study's relevance and clarity, as well as your constructive suggestions for further improvement.

Introduction
I would recommend adding some information about triggered EMA designs, since you are referring to these particular types of event-based EMA assessments.

>>> Thank you for your suggestion. We have adjusted the relevant section of the manuscript to clarify what triggered EMA designs are. The updated text now reads:

*Recent advances in sensor technologies have introduced the novel concept of event-based EMA, where surveys are dynamically triggered by specific events detected by sensors, offering a more context-aware alternative to traditional time-based approaches [21,22]. For instance, sensors embedded in wearable activity trackers can now detect prolonged bouts of sedentary behaviour [23] or episodes of walking and prompt participants to complete surveys based on these events [24].* *[lines 97 - 103]*

Furthermore, there are already studies on triggered EMA designs that have addressed specificity and sensitivity, which could strengthen your argumentation that we need

feasibility studies to “fine-tuning” the EMA-protocol.

>>> Thank you for your comment. We appreciate your observation and note that the importance of feasibility studies for fine-tuning EMA protocols, including addressing specificity and sensitivity, is already highlighted in the introduction. To further strengthen this argument, we have expanded the discussion section to incorporate additional context and references [Giurgiu et al., 2020; Kanning et al., 2022], as reflected in the updated text:

*"To ensure that participants receive approximately 4 surveys on sedentary behaviour, it was recommended that the duration of the sedentary episode be shortened from 30 to 20 minutes, consistent with durations used in other studies on sensor-triggered surveys of sedentary behaviour, which have employed either 20 or 30 minutes as thresholds to balance sensitivity and specificity [24]."* *[lines 639 - 643]*

*"While our study relied on a simple threshold in steps per minute to detect walking episodes, future studies might benefit from integrating additional data sources, such as GPS, as studies combining accelerometers and GPS have shown improved accuracy and richer contextual insights into walking behaviours [40]."* *[lines 670 - 674]*

Method
Provide some information about how many people you will need for your feasibility study and a rationale for how the number of participants will be distributed among the four different countries. The EMA-protocol varies by country, and this may be one reason for how many participants you recruit in each country. But that should be explained more clearly.

>>> Thank you for your suggestion. To address this, we have clarified the recruitment target in the manuscript. The updated Population section now includes the following sentence:

*"We aimed to recruit at least 10 participants from each country, totalling at least 50 participants."* *[lines 198 - 199]*

This target was chosen to ensure representation across the four countries while maintaining a feasible sample size for a pilot feasibility study.

Results
Please provide detailed information on the descriptives of the samples you combined or analyzed separately. For example, if feedback interviews were only conducted in Czechia and Germany, what was the gender distribution in these subsamples? Also, do you have information on BMI or the general volume of physical activity to further describe your sample in terms of key behaviors.

>>> We have included detailed per-country descriptives in the manuscript, including gender distribution, as follows:

*"A convenience sample of 52 participants (31±9 years, 56% females) from four different countries, France (n=11, 33±13 years, 27% females), Germany (n=12, 35±11 years, 75% females), Ireland (n=13, 29±7 years, 62% females), and Czechia (n=16, 28±6 years, 56% females), participated in the study. All participants completed the study."* *[lines 437 - 441]*

Unfortunately, we do not have additional descriptive characteristics, such as BMI, available for further describing the sample.

However, we calculated the average daily number of steps for 46 participants with 7 valid days of Fitbit data, as reported in the section "Event-based trigger optimisation":

*"Among these participants, the average daily wear time was 1,357±124 minutes, and the average daily step count was 11,189±2,881."* *[lines 470 - 471]*

Discussion
There are already some feasibility studies of EMA-protocols addressing compliance rate and burden of participants in EMA-studies that should be mentioned to integrate your findings into this area of research. There are also some studies on triggered e-diary assessments of sedentary behavior (e.g. JMIR mHealth and uHealth - Accuracy of Sedentary Behavior–Triggered Ecological Momentary Assessment for Collecting Contextual Information: Development and Feasibility Study) or walking behavior (e.g. JMIR Formative Research - Analyzing Person-Place Interactions During Walking Episodes: Innovative Ambulatory Assessment Approach of Walking-Triggered e-Diaries) that you could use to categorize and evaluate your findings (in section 2. Fine-tuning the rules for triggering the event-based surveys)

>>> Thank you for your insightful suggestion. In Section 2: "Fine-tuning the rules for triggering the event-based surveys," we have further contextualized our findings by discussing studies that explore triggered e-diary assessments of sedentary and walking behaviours [*Giurgiu et al., 2020; Kanning et al., 2022*], as reflected in the updated text:

*"To ensure that participants receive approximately 4 surveys on sedentary behaviour, it was recommended that the duration of the sedentary episode be shortened from 30 to 20 minutes, consistent with durations used in other studies on sensor-triggered surveys of sedentary behaviour, which have employed either 20 or 30 minutes as thresholds to balance sensitivity and specificity [24]."* *[lines 639 - 643]*

*"While our study relied on a simple threshold in steps per minute to detect walking episodes, future studies might benefit from integrating additional data sources, such as GPS, as studies combining accelerometers and GPS have shown improved accuracy and richer contextual insights into walking behaviours [39]."* *[lines 670 - 674]*

We believe this enhances the integration of our results into the broader literature.

You mentioned difficulties in assessing different physical activity behaviors (e.g. sedentary behavior, sleep). Although it is beyond the scope of your paper to provide a detailed validation study, you should discuss the advantages and disadvantages of using the Fitbit to achieve your goals of assessing different types of physical activity behaviors.

>>> Thank you for your thoughtful comment. While we agree that discussing the advantages and disadvantages of using Fitbit and other commercial devices, such as Garmin or Apple Watch, is important, we believe a detailed discussion of this topic extends beyond the scope of our paper, which focuses on the feasibility of the EMA protocol rather than a validation of specific devices. However, we acknowledge the relevance of this issue and note that some advantages and disadvantages of using Fitbit were already discussed in the Methods section, where we present the rationale for choosing this device:

*"Fitbit devices have been demonstrated to accurately detect steps in minute epochs, a necessary prerequisite for triggering event-based EMA surveys [30]. Additionally, Fitbit has been shown to be reasonably accurate in detecting episodes of sedentary behaviour [30]. However, a limitation of Fitbit is that the device automatically syncs with the Fitbit server only approximately every 15 minutes, resulting in delays in data evaluation. This delay, inherent to the Fitbit ecosystem, can be further prolonged by irregular syncs due to an unreliable internet connection or participants' smartphone usage preferences and behaviour." [lines 255 - 262]*

We appreciate your understanding and hope this addresses your concern.

Some participants received compensation, and some did not. Based on the results, compensation does not appear to have a significant effect on compliance. But you didn’t address this issue in your results. Because compensation is an important issue in conducting intensive longitudinal studies, I recommend addressing this issue in the Method section (e.g. provide some analysis of whether compensation influences compliance) and discussing it in the Discussion section. You also address this issue in your feedback interview. Perhaps you could add some qualitative statements about how participants feel about appropriate compensation.

>>> We appreciate your insightful comment. Incentives are undoubtedly a critical factor in conducting intensive longitudinal studies. However, given that the protocol was not strictly harmonised across the countries (e.g., different triggering rules, different approaches to meals, snacks, and drinks surveys), we are reluctant to draw substantial conclusions from the between-country comparison. Additionally, we do not have any qualitative data related to this topic. While reflecting on your comment, we recognised that this is an important limitation of our study and have added the following statement to the limitations section:

*"Fourth, the variability in participant compensation between countries (with incentives provided only in Ireland and Czechia) introduces a potential confound that could influence compliance rates. However, as the incentives were modest (20 euros), it is reasonable to assume they did not have a significant impact on compliance." [lines 707 - 711]*

We hope that this addition addresses your point.

**Reviewer #3:**

This was a very helpful and rigorous paper outlining challenges associated with EMA sampling schemes for health behaviors in free-living populations. The recommendations at the end are well-supported by the researchers’ findings and are an important contribution that will help to inform future research. I've included minor feedback/edits below:

>>> Thank you for your kind and encouraging feedback. We greatly appreciate your recognition of our work and your valuable suggestions to further enhance the manuscript.

Line 164: it is unclear what “target number” is referring to. Do the authors mean “maximal compliance” (i.e., 100% of triggered EMA are answered), or something else?

>>> Thank you for noticing this. We apologise for the confusion. The intended term was 'optimal number per day', which was introduced earlier in the text:

*"Another challenge specifically relates to event-based EMA, which requires fine-tuning the rules for detecting events of interest and triggering an optimal number of prompts...." [lines 137 - 139]*

The 'optimal number' is further explained in the Methods section as the number desired for the WEALTH project, specified as 4 for sedentary and 3 for walking surveys:

*"During the development of the EMA protocol, the WEALTH team also determined the optimal number of event-based surveys through expert consensus to align with the project objectives of collecting a large dataset of EMA-labeled accelerometry data for the application of machine learning methods. Specifically, the optimal number of surveys was set at four per day for sedentary surveys and three per day for walking and running surveys, balancing the feasibility of collecting sufficient data with minimizing participant burden." [lines 285 - 291]*

In addition to updating line 164, we have also replaced the term 'target number' at two other places *[lines 285 and 289]* in the manuscript to ensure consistency. We hope this clarification resolves the issue.

Lines 192-196: why were participants not screened for habitual activity levels to ensure that at least some vigorous activity occurred, if they were asked to perform their usual behavior? Also, did they screen for mobile data plan?

>>> Participants were not screened for habitual activity levels to simplify the recruitment process; however, we acknowledge that doing so might have improved the detection of running events in the study. However, we did screen participants for having a mobile data plan, and we have updated the manuscript to reflect this:

*"Additionally, participants were required to be willing to carry and use their smartphones with a mobile data plan and engage in the entire data collection process throughout the study period." [lines 203 - 205]*

We appreciate your observation and hope this clarification addresses your concern.

Line 251-252: The authors mention that lack of internet connectivity can interfere with Fitbit syncing. Were data not uploaded using cellular data—WiFi only? And could event-contingent EMA only be received when WiFi present, or could they also be received via cellular data?
>>> Thank you for your comment. The data could be uploaded using both cellular data and WiFi. We believe this is now clearer with the introduction of the mobile data plan screening, as noted in our response to the previous comment. Additionally, the use of cellular data is explicitly addressed in the Procedures section of the manuscript:

*"Additionally, they were instructed to adjust their smartphone settings (mobile data always on, unmute or at least set to vibration, no battery saving mode, Fitbit notifications off, HealthReact notifications on) and to maintain these settings throughout the study." [lines 358 - 361]*

Also-- Were participants asked to wear Fitbit overnight? And for how long during the day?

>>> Thank you for noticing this. Participants were required to wear the Fitbit 24 hours a day throughout the entire study period, with the exception of one night designated for recharging the device. We have clarified this requirement in the manuscript:

*"Before leaving the lab, participants were asked to maintain their usual behaviour throughout the study, wear the Fitbit 24 hours a day (except for one night designated for recharging the device), report all their meals, snacks, and drinks, and make every effort to complete as many prompted surveys as possible." [lines 367 - 370]*

Can the authors provide data on average wear time, step count, SB volume, and walking volume from the Fitbit?

>>> We appreciate your comment. The average wear time and daily number of steps were calculated for 46 participants with 7 valid days of Fitbit data:

*"Among these participants, the average daily wear time was 1,357±124 minutes, and the average daily step count was 11,189±2,881." [lines 470 - 471]*

However, we are unable to provide data on sedentary behaviour volume, or walking volume.

Line 277: What do the researchers mean by a “lead-in day”?

>>> A “lead-in day” refers to a day used to allow participants to familiarize themselves with the study protocol and devices in their natural environment, ensuring they are comfortable with the procedures before the formal data collection begins. We have clarified this term in the manuscript:

*"The first day (day -1) served as a training day in the lab, the second day was a lead-in day (day 0) where participants familiarized themselves with the study protocol and devices in their natural environment, and the data from the subsequent 7 days were included in the analyses (days 1 to 7)." [lines 293 - 296]*

Line 381: “valid day required at least 10 hours with at least one heart rate recording”—do the authors mean to say “one heart rate recording per minute”? Otherwise, why is the criteria for wear time so low wrt heart rate; and 10 h of what type of data (e.g., nonzero triaxial readings)?

>>> To clarify, a valid day was defined as having at least 600 minutes (10 hours) where there was at least one heart rate recording logged for each of those minutes. We have adjusted the manuscript to reflect this definition:

*"... only data from participants who had a full 7 valid days were used, where a valid day was defined as having at least 600 minutes (10 hours) where there was at least one heart rate recording logged for each of those minutes." [lines 402 - 404]*

A table with participant characteristics at baseline would be helpful.
>>> Unfortunately, we do not have participant characteristics beyond age and sex, which are already included in the text, so adding an additional table would not provide substantial value. However, since the results throughout the manuscript are reported at the level of individual countries, we find it useful to report age and sex by country, as also suggested by Reviewer 2. The updated text now reads:

*"A convenience sample of 52 participants (31±9 years, 56% females) from four different countries, France (n=11, 33±13 years, 27% females), Germany (n=12, 35±11 years, 75% females), Ireland (n=13, 29±7 years, 62% females), and Czechia (n=16, 28±6 years, 56% females), participated in the study." [lines 437 - 441]*

Lines 417-418: Could this also be due to a lack of running activity in the sample (i.e., sample is fairly inactive), rather than the triggering rules?

>>> Thank you for your insightful comment. The number of detected events is influenced by multiple factors: participants’ behaviour (time spent running), event definition (triggering rules), and data availability (wear time and internet connection). In the entire dataset, there were 795 minutes with 140 or more steps per minute, indicative of running. However, the number of running events is not solely dependent on the total time spent running but also on the pattern of running sessions. For example, runs shorter than 5 or 10 minutes would not trigger any event under the current rules. Similarly, a single ultramarathon lasting 795 minutes would only trigger one event. Adjusting the triggering rules, such as decreasing the minimum interval between two triggers from 90 to 5 minutes, would increase the number of detected events but could significantly increase the burden on participants, potentially triggering a survey every 5 minutes during a longer run. We hope this explanation clarifies the observed very low number of running triggers.

Lines 445-446: How did the authors decide on the “optimal number of surveys” for the project? It’s a little unclear how these target numbers were chosen.

>>> We appreciate your question. The optimal number of surveys was determined through an expert consensus by the multidisciplinary WEALTH team to align with the project’s objectives. These objectives focused on collecting a large dataset of EMA-labeled accelerometry data for the application of machine learning methods while considering the burden on participants. We have updated the manuscript accordingly:

*"During the development of the EMA protocol, the WEALTH team also determined the optimal number of event-based surveys through expert consensus to align with the project objectives of collecting a large dataset of EMA-labeled accelerometry data for the application of machine learning methods. Specifically, the optimal number of surveys was set at four per day for sedentary surveys and three per day for walking and running surveys, balancing the feasibility of collecting sufficient data with minimizing participant burden." [lines 285 - 291]*

Also, did the authors determine the degree of sensitivity & specificity associated with the optimal settings (I assume EMA response data captured self-reported activity that could be used to assess this)?

>>> We appreciate your thoughtful question. While we did capture self-reported activity, we are reluctant to consider it as the ground truth for comparing Fitbit-detected events to determine sensitivity and specificity. We agree that it would be interesting to correlate Fitbit-detected and participant-reported physical behaviours; however, this falls outside the scope of the current manuscript.

Page 30, Improving participants’ compliance section: Other possible recommendations could include a “snooze” feature (e.g., option to silence and delay the prompt for another 30 min, like an alarm clock), or possibly just more monetary incentive tied to EMA response rates (e.g., graded compensation based on percentage of EMA completed).

>>> We appreciate your suggestions. The potential impact of a snooze feature and graded compensation on compliance has been considered, and we have addressed these points in the revised manuscript:

*"Similarly, introducing a snooze feature that allows participants to delay the survey could improve compliance by offering additional flexibility; however, this approach would result in responses being even further removed from the event of interest, which is why we did not recommend it." [lines 629 - 632]*

*"Future studies should explore the impact of incentives in a more rigorous way, for example, by using graded compensation tied to EMA response rates, to further enhance participant compliance." [lines 711 - 713]*

Line 605: reference needs to be fixed.

>>> Thank you. The reference has been fixed [Stamatakis et al., 2019].

Limitations section should include the use of convenience sampling from existing social networks (might lead to greater social desirability bias during exit interviews). Also, the study provided only a small compensation (20 euros) for some participants (Ireland and Czechia), whereas others had no monetary incentive, and this might have contributed to the low compliance rate.

>>> Thank you for highlighting these important points, which coincide with feedback from another reviewer. We have included these considerations in the limitations section, and the updated text now reads:

*"Fourth, the variability in participant compensation between countries (with incentives provided only in Ireland and Czechia) introduces a potential confound that could influence compliance rates. However, as the incentives were modest (20 euros), it is reasonable to assume they did not have a significant impact on compliance." [lines 707 - 711]*

*"Finally, the recruitment of a convenience sample, including individuals personally familiar to the authors, may have influenced participants' motivation to complete the study and contributed to the unusually high retention rate due to a sense of interpersonal obligation. [lines 714 - 718]*

We value your input in refining the clarity and scope of our manuscript.
